# Supplementary material for: Clustering of HR + /HER2− breast cancer in an Asian cohort is driven by immune phenotypes
Source: Breast Cancer Res. 2024 Apr 22;26:67. doi: 10.1186/s13058-024-01826-5 (PMC11035138; doi:10.1186/s13058-024-01826-5)
Supplement: Supplementary file 1 — Additional file 1. Supplementary Figures S1–S4. [file 13058_2024_1826_MOESM1_ESM.pdf]

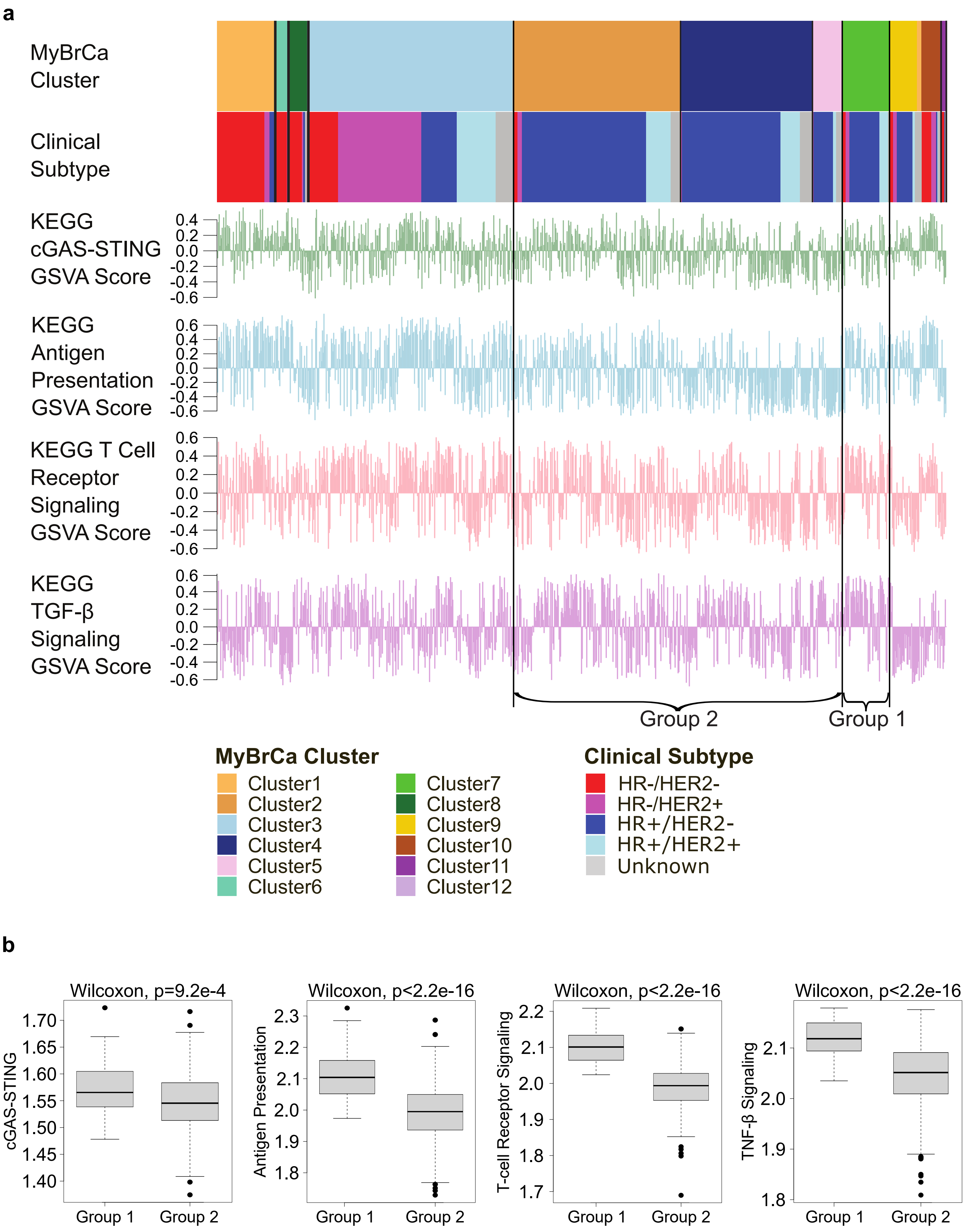

Supplementary Figure 1 (a) Comparison of MyBrCa Clusters and clinical subtypes with KEGG pathway GSVA scores. HR+ clusters with high immune scores (Group 1) and low immune scores (Group 2) are indicated. **(b)** Comparison of KEGG pathways GSEA scores between Group 1 and Group 2.

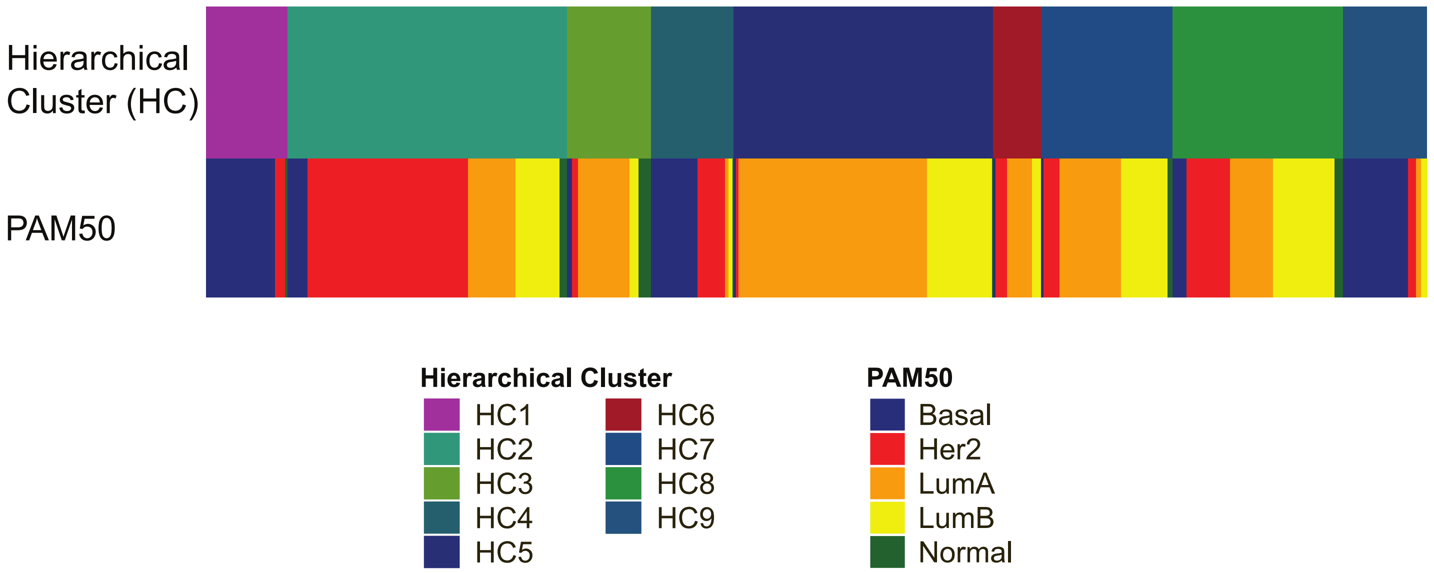

Supplementary Figure 2 Comparison of clustering results of the MyBrCa cohort using hierarchical clustering (HC) and PAM50.

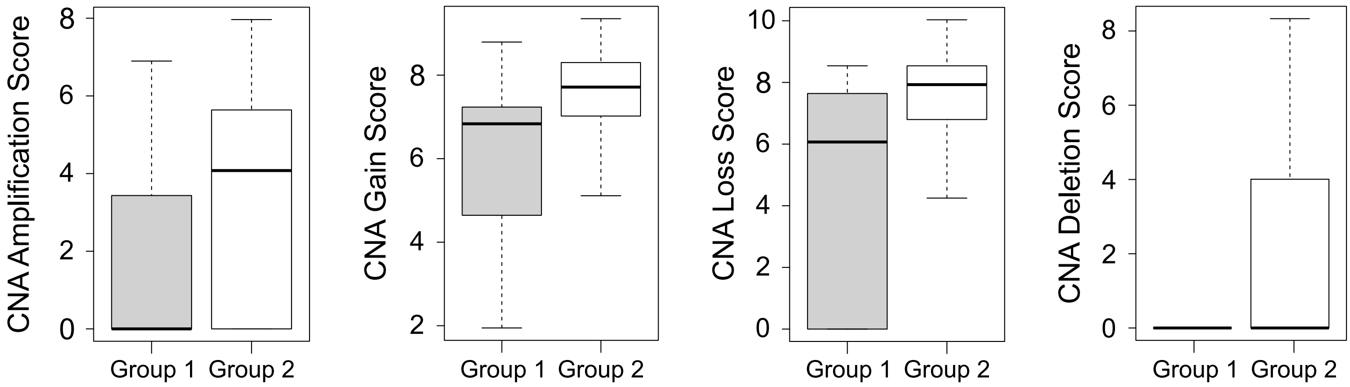

Supplementary Figure 3 Comparison of Copy Number Aberration (CNA) amplification, gain, loss and deletion scores between Group 1 and Group 2.

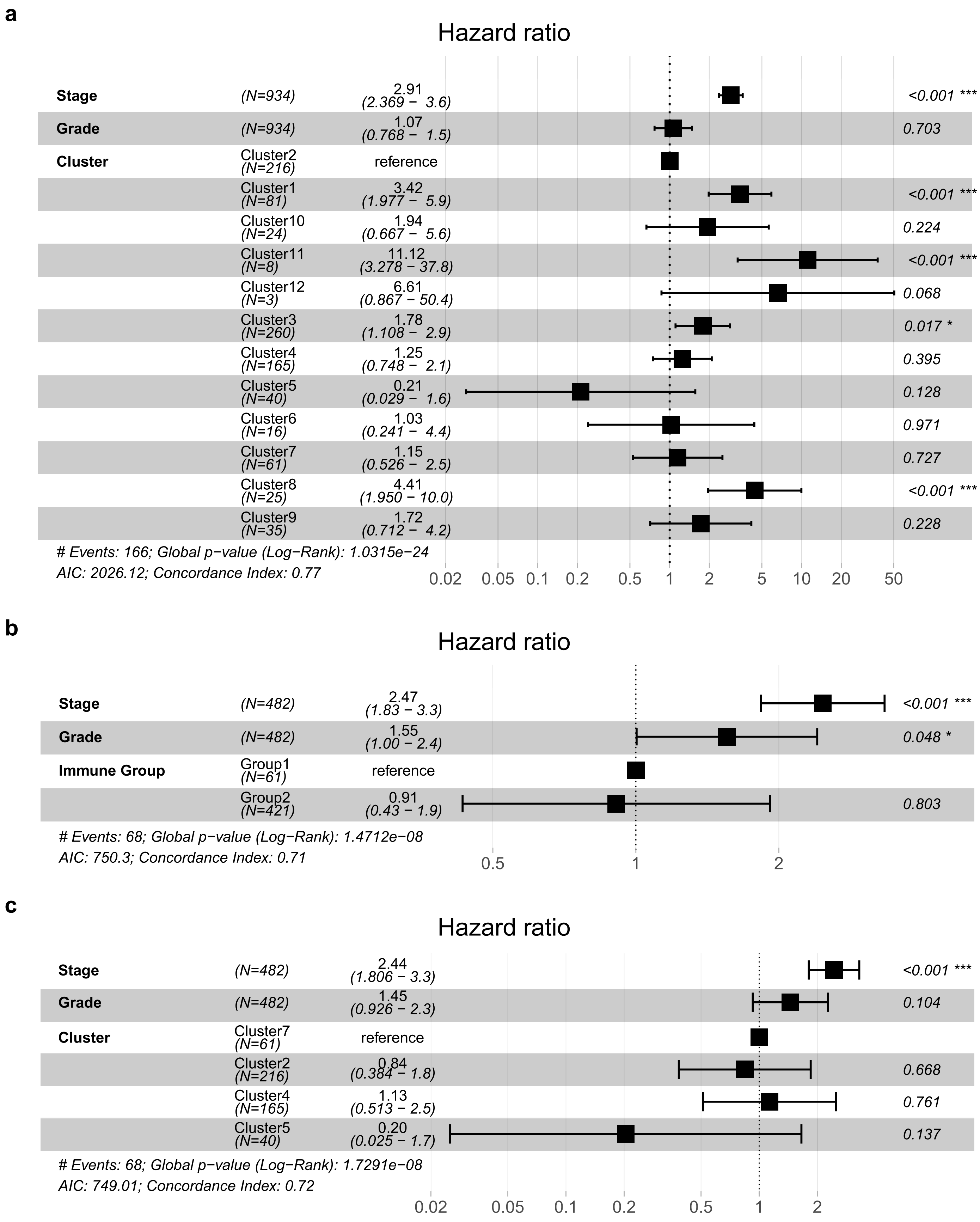

Supplementary Figure 4 Cox proportional hazard models of overall survival of MyBrCa patients. (a) Stage, grade and clusters were included as variables. **(b)** Stage, grade and immune group were included as variables. **(c)** Stage, grade and clusters were included as variables, but only clusters belonging to Group 1 and Group 2 were included. Error bars represent 95% confidence interval of hazard ratio.
